# Current Nursing Education Considering Southern Europe’s Reality and Legal Framework: A Two-Phased Research Approach

**DOI:** 10.3390/nursrep13040113

**Published:** 2023-09-28

**Authors:** Celeste Antão, Aloma Antolí-Forner, Hélder Fernandes, Sara Brás Alves, Helena Pimentel

**Affiliations:** 1 Instituto Politécnico de Bragança, Campus de Santa Apolónia, 5300-253 Bragança, Portugal; helder@ipb.pt (H.F.); sarabras@ipb.pt (S.B.A.); hpimentel@ipb.pt (H.P.); 2 Health Sciences Research Unit: Nursing (UICISA: E), Health School of Bragança, Avenida D. Afonso V, 5300-121 Bragança, Portugal; 3 Department of Nursing, Universitat Jaume I, Avenida de Vicent Sos Baynat, 12071 Castelló, Spain; al404154@uji.es

**Keywords:** nursing curriculum, degree, quality

## Abstract

Nursing education and formation is still heterogenous in spite of The Bologna Declaration in 1999. Apart from the existence of basic curriculum standards, universities have flexibility regarding its design. The objective of this study is to provide an overview of contemporary nursing education and contrast it with the legal frameworks in place in four Southern European countries: Portugal, Spain, Italy, and Greece. A scoping review was conducted in order to revise and obtain an up-to-date review of current nursing education and quality. Then, a content evaluation of the legal public framework was conducted. A total of nine articles were included in the review. Data analysis evoked three main themes from the results: nursing education heterogeneity owing to ECTS increased from 180 to 240 for the diversity of clinical practice hours; the nursing framework lacked a definition; and the evolution of nursing education, alongside quality improvement and more accurate guidelines were required. Regarding their legal framework, the main directives and legislation standards were assessed and compared with the current curriculum. To conclude, nursing heterogeneity education evokes competence inequality among students and future professionals as larger curricular programs (240 ECTS) offer more clinical practice. Nursing education uniformity could enhance international mobility and promote knowledge exchange and nursing competence framework definitions. These are facts that certainly bring nursing empowerment. This study was not registered.

## 1. Introduction

In 1999, 29 European countries joined The Bologna Declaration, identified as an agreement meant to enhance cohesion and homogeneity in higher education in Europe. Thus, all members agreed to move toward the introduction of a three-cycle higher education frame, a qualification recognition abroad method and a quality of the assurance system [1,2]. Currently, 49 countries and the European Commission constitute The Bologna Follow-Up Group [3].

The main focus of the Bologna Process was the foundation of the European Higher Education Area (EHEA) [2]. Furthermore, the constitution of the European Credit Transfer and Accumulation System (ECTS) and the Diploma Supplement were measures adopted within the action plan. Students represent a central role in the new conception of ECTS, measuring learning outcomes and curricular workload via a system of credits. A full-time academic year is represented by 60 ECTS, and one credit corresponds to 25–30 h of work [4]. The Diploma Supplement consists of a document attached to the higher education diploma, which highlights curriculum content and learning achievement [5].

Regarding professional qualifications and regulations, the 2013/55/EU directive of the European Parliament [6] established the requirements of the common training framework in order to offer equity in education and define professional competencies. Despite the homogeneity endorsed by the Bologna Process, the literature still states that there is a variety between nursing curriculum programs in Europe due to cultural and knowledge inequalities [6,7]. Moreover, this heterogeneity leads to a difficult definition of the nursing role [8], discouraging international mobility [7].

According to recent studies, new concepts have to be considered to develop a transnational nursing curriculum and to build a more global higher education nursing approach. More creativity, communication, quality monitoring, and consideration of students’ experiences are required to ensure successful education and reduce cultural and social diversity [9]. Language and communication difficulties, prejudices and stereotypes, and lack of cultural competence training were identified as challenges to nursing in a clinical framework [10]. However, communication skills training development in the educational curriculum could prevent such difficulties and increase the quality of health care [11].

Therefore, the present study aims to bring an overview of current nursing education and compare the results with the legal framework involved among four southern European countries: Portugal, Spain, Italy, and Greece.

## 2. Materials and Methods

The study methodology followed a two-phase research approach in order to answer our research question: What is the current state of nursing education focusing on curriculum and legal variations among four European countries? First, a scoping review was conducted to revise and obtain an up-to-date assessment of current nursing education and quality. By including studies from other continents, it was expected to confirm heterogeneity in nursing education over the world and to determine other education challenges. Afterward, a content evaluation of the legal public framework written for higher education nursing was realized within the four selected countries. Therefore, it was intended to make a comparison between the scoping review results and the specific legal framework applied to the countries selected.

The selection of these countries was made aiming to evaluate the situation in Southern Europe and focus the analysis and comparison within a specific region. Southern European countries, where the authors of the article work, both have a Latin-speaking origin, are geographically close, and have some cultural similarities, thus creating an opportunity for the mobility of nursing students, boosted by the ERASMUS+ program.

Consequently, addressing the main distinctions between these countries would be advantageous in promoting student mobility, as it could help reduce and resolve many of the difficulties often encountered due to variations in curricula and ECTS validation.

### 2.1. Scoping Review Eligibility Criteria, Information Sources and Search Strategy

The scoping review phase was conducted following the Joanna Briggs Institute methodology for scoping reviews [12] since it is identified as a rigorous and updated methodological procedure to procure high-quality reviews [13]. The Preferred Reporting Items for Systematic Reviews and Meta-Analyses extension for Scoping Reviews (PRISMA-ScR) checklist and explanation were used once this methodology improved research quality and reliability [14]. This guide has the purpose of promoting more transparent, complete, and accurate reviews of the literature and to ease evidence-based decision making [15].

To generate the research question and inclusion/exclusion criteria, the PCC (Population, Concept, and Context) framework was applied and fulfilled with JBI guidance [16] (Table 1).

The PCC model helped to construct the general research question for this research, as well as the specific eligibility criteria established. The population of interest were ungraduated and graduated nurses. This concept addressed the nursing degree and quality field, and the context was current nursing education around the globe.

The review included primary studies, such as observational, descriptive, mixed-methods, quantitative, and qualitative. Only studies published within the last 10 years in English, Portuguese, or Spanish languages were included. Several factors were considered in the decision to limit the focus of this review to studies conducted within the past 10 years. Education evolves constantly; therefore, focusing on the literature published recently allowed us to find the latest advancements, education innovations, and the most recent research in the field.

This review was conducted in July 2023 utilizing the following databases: Web of Science, PubMed, and b-On. The search strategy included a combination of keywords with the Boolean operator AND to identify articles relevant to the topic. “Nursing education AND degree AND quality” composed the search’s syntax structure. The included studies were further analyzed following the identified emerging themes.

### 2.2. Scoping Review Study Selection Process, Data Collection Process, Extraction and Analysis

The present search and data selection were conducted by two independent reviewers (AAF and SA) through the selection of scientific databases and evaluation and eligibility of studies, which started by analyzing the title and abstract. Afterward, the full-text paper was screened using AAF and reviewed by S to verify if the studies met the inclusion criteria. If discrepancies between reviewers occurred regarding the eligibility, inclusion, or not of these studies, they were solved through the consultation of three independent reviewers (HP, HF, and CA) to achieve an agreement. Records were managed via Rayyan, an online tool for systematic reviews, to assess study selection [17], and Mendeley, a specific software for managing bibliographies. For each study, an electronic table containing the following topics was obtained: authorship, year of publication, location, sample size, study design, main goal, outcomes, and key findings (Table 2). Considering the small number of studies, a narrative synthesis was carried out as part of the results of the present review.

### 2.3. Legal Document Evaluation and Framework Evaluation

Legal public documents about European Nursing Education were retrieved following two stages. First, an identification of relevant European Union Websites was conducted to obtain the legal reports. The main web pages consulted were the European Commission [2], the European Higher Education Area [3], and the European Union’s official law portal (EUR-Lex) [18]. Additionally, a search by country was realized, including Portugal, Spain, Italy, and Greece’s legal framework. Their specific area inside the European Union web pages [19,20,21,22] was consulted. Afterward, the search was focused on the directives and regulations of each country, which provided specific country nursing education and legal contexts. Specific and relevant keywords such as “nursing degree legislation” and “nursing education regulation” were used to access goal documents.

In relation to the language, papers written in Portuguese, English, or Spanish were assessed. However, legal documents in Greek were not able to be analyzed due to the language barrier.

## 3. Results

### 3.1. Scoping Review

**Figure 1:**
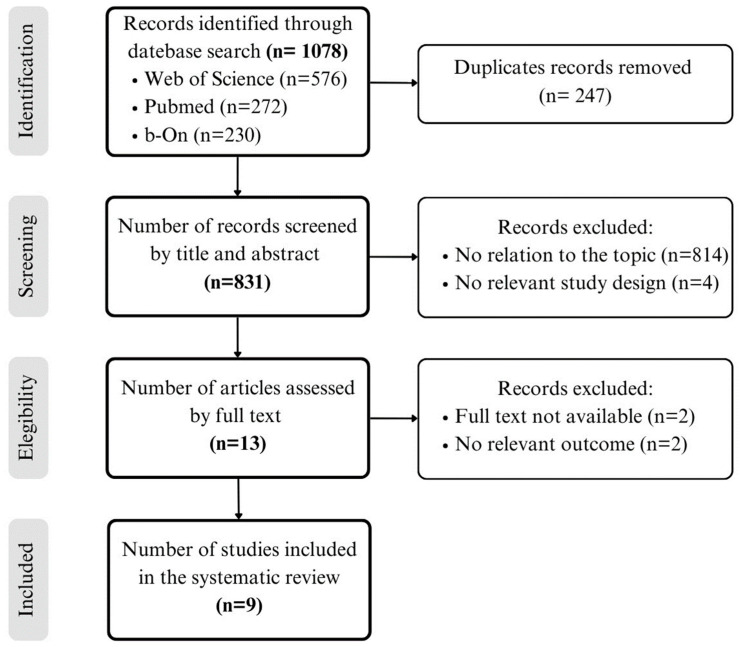
PRISMA flowchart and selection process. Source: own elaboration.

The initial search identified 1078 records. After removing the duplicates, 831 articles were screened by title and abstract to assess their relevance according to the proposed inclusion/exclusion criteria. Subsequently, the full text of 13 articles was reviewed to determine if they met the eligibility criteria. The Preferred Reporting Items for Systematic Reviews and Meta-Analysis (PRISMA) diagram outlines the review process and search outcomes [23] (Figure 1). A total of nine studies were finally included in the review (Table 3).

All articles included were scientific studies published in different nursing journals. In relation to the design of the studies selected, quantitative (n = 4), qualitative (n = 2), and mixed-method (n = 3) approaches were identified. Regarding the quantitative studies, two followed a cross-sectional design [24,25], and two were descriptive [26,27]. In relation to qualitative ones, one was a review of the literature with a focus group constitution [28], and the other underwent quality framework appraisal [29]. According to the mixed-method studies incorporated, two were reviews of the literature, including empirical research [30,31], and the last one had a mixed qualitative and quantitative approach [32]. Concerning the origin of these studies, almost half of them addressed nursing education in Europe (n = 4) [25,28,30,32]. The rest tackled nursing education in North America (n = 3) [27,29,31], South America [26], among East and Southeast Asia [24]. The majority of these studies reported the current situation of the nursing curricular degree and its evaluation [26,27,28,29,31]. Two studies addressed nursing competence areas and nursing clinical frameworks [25,30], and the last two analyzed specialization and doctoral programs [24,32].

**Table 3:** Summary of included articles.

Authors (Year) and Country	Sample Size	Study Design	Main Goal	Outcomes	Key Findings
Satu et al. (2013) Finland [30]	--	Review of the literature and European documents search	To identify nursing competence areas in Europe.	8 main categories of nursing competence areas were identified.	Determining common nursing competence areas within Europe would promote nurse mobility and uniform nursing education.
Molassiotis et al. (2020) Hong Kong [24]	20 coordinators and 135 doctoral students	Cross-sectional	To describe East and Southeast Asian nursing doctoral characteristics and to explore doctoral student’s experiences.	Identified challenges: faculty shortage, delays in the completion of doctoral programs, and inadequate financial support. High prevalence in supervision satisfaction, paper publishing pressure, and curriculum satisfaction. Mobility opportunities were not included in around 50% of student programs.	Positive experiences are generally held by students. More international mobility opportunities are required. International guidelines and quality doctoral program indicators are also needed to deal with global nursing education challenges.
Kiekkas et al. (2019) Greece [25]	285 nursing students	Cross-sectional	To investigate self-reported competence factors associated and educational issues perceived in Greek nursing students.	Self-reported competence was identified to be good. A significant association between self-reported competence and quality program perception was outlined.	The outcomes defended the improvement program’s quality and importance. The increase in clinical training hours and the use of critical thinking are supported.
Meira and Kurcgant (2016) Brazil [26]	19 nursing graduates 15 employers 5 teachers—Focus group	Descriptive and exploratory study	To support the assessment and changes in the nursing curriculum.	An action plan was created as a curriculum quality improvement example. Suggestions involved were curriculum flexibility, content resizing, continuing education, practice enhancement, active methodologies, and the autonomy of the student.	The current context of nursing education and curriculum demands were identified. This fact promoted the Action plan elaboration to deal with the improvement of the nursing curriculum.
Ruiz-Rojo et al. (2022) Spain [28]	60 Spanish nursing curriculums	Mixed-methods approach	To explore differences in nursing degrees, to compare results with legislation, and to propose changes.	Differences between public and private universities were statistically significant. Most of the Legislation criteria was accomplished by the academic curriculum. Standardization curricula are proposed.	There is high heterogeneity between the Spanish nursing curricula. The homogeneity of teaching blocks between universities promotes student mobility and nursing degree standardization.
Tavernier and Wolf (2022) USA [29]	49 students and faculty members	Qualitative explorative descriptive	To handle limitations in quality improvement strategies, to involve students, and to execute real-time changes.	The analysis of the Committee created defined three aspects to consider: personal and professional impacts, facilitators to participation, and barriers to participation.	Student participation is a continuous quality improvement program that brings the chance to collaborate with the faculty on important issues and to obtain essential skills such as leadership, among others.
Decock et al. (2022) France, Croatia, Belgium [32]	40 organization leaders 498 nurses	Mixed-methods study. Quantitative and qualitative approach.	To recognize the interfaces of the specialist nurse profession across the EU.	Countries’ characteristics, educational level, autonomy and responsibility, suggestions, and qualitative research results were analyzed.	Homogeneity is required to define a specialist nurse’s role. This fact promotes the advanced practice of nursing and mobility around the EU.
Jager et al. (2020) Canada [31]	--	Integrative review and empirical research	To identify existing curriculum renewal strategies, to use an evidence-informed process, and to discuss the nursing curriculum and the ever-changing healthcare context.	The Ottawa model for nursing was identified as a model procedure for nurse curriculum renewal.	Help in defining the nursing curriculum renewal procedure was provided with this analysis. The Ottawa model is useful for attempting nursing curriculum renewal.
Cipher et al. (2021) USA [27]	271 nursing students	Descriptive comparative study	To identify clinical and simulation hours of 4 nursing programs.	Substantial variability in the number of clinical and simulation hours was identified between the 4 programs (ranging from 796 to 948 h).	These outcomes propose that the student’s evaluation is not equivalent to the hours of practice. Clinical requirements based on empirical evidence are suggested for future decisions.

Source: own elaboration.

### 3.2. Legal Framework

Concerning the European legislation about higher education, Directive 2013/55EU [6] was the main public document to procure an overview of the current legal bases and standards meant to be applied. The European Union reported that nursing degrees must comprehend at least three years of study measured with ECTS, representing no less than 4600 h of theoretical and clinical training. The minimum duration of the theoretical component has to be at least one-third of the workload, and at least one-half of the curriculum ECTS has to be designated for clinical training [6].

The legal framework applied in Portugal [33,34,35], Spain [36,37,38,39,40], Italy [41,42,43], and Greece [44,45,46,47] was also identified. The four countries joined The Bologna Declaration in 1999 [3]. Higher education in these countries adopted ECTS, a Supplement Diploma, and a three-cycle studies organization as follows: study programs (degree), postgraduate programs, and doctoral programs. A full academic year was represented with 60 ECTS in the four countries [20,34,36,41].

Spanish legislation describes a distribution of nursing learning contents in three blocks of common core education (60 ECTS), nursing sciences (60 ECTS), supervised internships/thesis (90 ECTS), and elective subjects (30 ECTS) [36]. The recent Law 2/2023 [38] regulates universities’ autonomy to adapt and organize the structure and contents of their educational programs. Regarding Italy, they also divide their contents into four blocks of basic subjects, characteristic subjects, relational subjects, and other subjects, which include elective subjects, the final practice exam, and the final degree’s dissertation. The amount of ECTS corresponding to each block can vary between universities; however, clinical practice is settled to involve a minimum of 60 ECTSs [41]. Portuguese legal bases do not present a curricular content distribution defined but establish a practical component comprehending at least half of the course workload [48]. In relation to Greece, a specific nursing curriculum’s legal basis could not be assessed due to the language barrier and the inexistence of official translations. However, the web page of the “Nursing School Ateith” described their curricular program. Their degree consisted of 48 subjects: 39 compulsory, 12 electives, and 3 obligatory free choices. Their course structure is divided into 4 groups: basic science division, basic nursing division, nursing specialties division, and humanities and social division [49].

## 4. Discussion

The aims of this review were to provide an overview of the present status of nursing education and to conduct a comparative study of the findings with the legal framework involved in four southern European countries: Portugal, Spain, Italy, and Greece.

The results of the scoping review led to three primary themes for assessment, while the legal framework evaluation uncovered the fundamental legal foundations pertaining to the nursing curriculum within selected countries. The mentioned principal topics extracted from the results were:

- Nursing education heterogeneity;
- Nursing clinical framework and competence areas poorly defined;
- Nursing education evolution, quality improvement, guidelines, and standard requirements.

First, the vast majority of the reviewed studies specified heterogeneity in nursing education. Ruiz-Rojo et al. [28], who analyzed the entire nursing degree curriculum in Spain, claimed differences between public and private universities according to subjects and ECTS distribution. Their study showed variability among nursing curriculums within the same country. This fact demands and requires the establishment of a more restrictive legislation basis according to the ECTS distribution and learning blocks definition. If there is a clear disparity in the content curriculum of one country, this heterogeneity can grow exponentially among international programs. Furthermore, in relation to clinical practice, Cipher et al. [28] described variances as well, suggesting to not examine results considering only the number of hours but also the empirical outcomes.

Moreover, nursing education heterogeneity is not only present in the degree but also in a specialist [32] and doctoral programs [24]. Regarding specialist nurses, Decock et al. [32] reported a lack of definition for the role of a specialist nurse, as well as a disparity in the process and training to become specialist in European countries. With respect to doctoral programs, Molassiotis et al. [24] claimed the establishment of guidelines and quality enhancement in order to guarantee doctoral programs; progress and development.

Furthermore, these last results outline the importance of defining a nurse’s role as it presents a lack of clear description, including a poor concept of nursing competence areas. Satu et al. [30] suggested that the establishment of common curriculum competence areas Europe could promote equality in nursing education, as well as assurances of high-quality nursing care. In addition, Kiekkas et al. [25] showed a positive association between nursing students with self-reported competence and perceived quality degree. In addition, the increment of clinical hours and promotion of critical thinking skills are proposed to enhance higher competence.

According to the general curriculum’s heterogeneity and the nurse’s clinical framework, findings from this study are also reflected in the existing literature. Kraaij et al. [8] reported that an international description of the nurse’s role is required in order to reduce nursing educational pathways and titles/degree diversity. Moreover, this variety between educational programs could lead to people’s application for the same job without the same level of education and not being able to fulfill the same role. Making the nursing curriculum uniform and standardized could promote international mobility between undergraduate and graduate nursing professionals.

Additionally, nurses are expected to respond to every clinical environment and to deal with a constantly changing society’s necessities with success. In order to handle people’s health needs, nursing education must be adapted and evolve as fast as society demands. Jager et al. [31] proposed The Ottawa Model as a proper option to address a curriculum renewal process and reported, as well as Meira and Kurcgant [26], that evaluation is the main aspect in order to manage a renewal procedure. Another consideration in relation to quality improvement is student procedure involvement. This fact could enhance their professional communication and problem-solving skills [29]. More accurate and restrictive guidelines and standard establishment are also identified as a required measure to enhance education quality [24,27,28,30,31,32].

According to the main European directives, Portugal, Spain, Greece, and Italy comply with the minimum year’s duration and the total compendium of hours established. Currently, the four selected countries include a bachelor’s degree in nursing within their higher education programs. However, in relation to the degree’s structure, whereas the nursing curriculum degree in Portugal, Spain, and Greece is constituted of 240 ECTS [36,48,49] distributed in four years of study, the nursing bachelor in Italy only comprehends 180 ECTSs distributed over three years of study [41]. Consequently, the main existing difference between these four countries studied is the duration of the degree and ECTS distribution. The present variety in Europe increases inequality between nursing students’ competence acquirement. The nursing curricula of the 240 ECTSs present require further ECTSs to be distributed for clinical practice.

European nursing and higher education legislation seems to be too lax. Considering the outlines of the present results, there is a clear requirement to establish more restrictive nursing uniformity bases [8,28]. Efforts affording nursing homogeneity could enhance the international mobility between undergraduates and graduates, as well as scientific knowledge exchange. The homogenization of nursing in healthcare could improve the quality of healthcare and simultaneously guarantee patient security, as they must be the mainstay of all care plans.

### 4.1. Study Implications

The present study provides an overview of the current heterogenous state of nursing education and the main differences between the curriculum legal bases of four Southern European Countries. The survey findings include data and evidence that support legal concerns or issues that need to be addressed regarding the nursing curriculum its more accurate regulation.

### 4.2. Study Limitations

The analysis realized in the investigation encountered several limitations, primarily due to the scarcity of studies in this area, especially those employing the same methodological approach. Additionally, despite the legislative efforts to promote standardized education and, consequently, professional practice, we acknowledge that gaps persist in terms of the number of ECTS credits and the duration of academic training required in terms of the number of years. Furthermore, in addition to nursing having a rich historical legacy as a profession, a notable disconnection persists among legislative frameworks, educational approaches, and professional practices. Such a situation can be improved through the coordination of these three essential components, among others. It is important to note that our results only address generic aspects of the educational landscape. Nevertheless, they can serve as a valuable starting point for a more in-depth and, if possible, more comprehensive analysis.

The language barrier was also a challenge to understand and analyze the Greek legislation, reports, and directives.

### 4.3. Future Research Directions

Future research is needed to justify and initiate legal action changes in relation to nursing education for the purpose of ensuring international uniformity.

## 5. Conclusions

The evidence presented strongly suggests that nursing heterogeneity education evokes competence inequality among students and future professionals. For instance, larger curricular programs (240 ECTS) offer more clinical practice. The call for nursing education uniformity becomes evident as it holds the potential to foster international mobility, promote knowledge exchange, and facilitate nursing competence framework definition. These facts certainly bring nursing empowerment and significant contributions to the nursing field on a global scale.

Two principal strategies are proposed to address the challenges identified in the study’s findings:

- The establishment of a comprehensive framework for elementary nursing education with a more precise delineation of nursing competencies
- The implementation of a standardized the global curriculum for nursing degrees and an equitable distribution of ECTSs.

These proposals represent an opportunity to enhance and advance nursing skills, encourage specialization, and promote the growth and development of doctoral education in the field. It is crucial to note that this second recommendation is intricately linked to the first. The achievement of the second proposal is contingent upon the successful realization of the first.

A more restrictive legal framework is required in order to accomplish nursing competence equality and to solve global curriculum uniformity shortage. It should be imperative that nursing professionals, regardless of their geographical location, possess uniform competencies to ensure patient’s safety and healthcare rights. This fact is underscored by the European Parliament in Directive 2011/24/EU, which emphasizes that patient’s cross-border healthcare should maintain quality attention. This legislation ensures patient mobility and contributes to social cohesion, social justice, and sustainable development.

## Figures and Tables

**Table 1:** PCC framework.

PCC	Eligibility Criteria
Population	Nursing students
Concept	Nursing degree and quality
Context	Nursing education

Source: own elaboration.

**Table 2:** Items extracted for data analysis.

Data	Study Characteristics	Main Conclusions
Publication date	Design	Outcomes
Author(s) information	Inclusion/exclusion criteria	Key findings
Location		
Participants		

Source: Own elaboration.

## Data Availability

Not applicable.

## Public Involvement Statement

There was no public involvement in any aspect of this research.

## Guidelines and Standards Statement

This manuscript was drafted against the Joanna Briggs Institute methodology for scoping reviews research.
